# 

**Published:** 1917-04

**Authors:** 


### OLD JOKES THAT WE HAVE MET.

"In the days of old Rameses that story had pareses." —*Kipling.*

A New Complaint.—A German, whose wife was ill at the Seney Hospital, Brooklyn, called the first evening she was there and inquired how she was getting along. He was told that she was improving.

Next day he called again, and was told she was still improving. This went on for some time, each day the report being that his wife was improving.

Finally, one night when he called he was told that his wife was dead. Seeing the doctor, he went up to him and said, with a world of sarcasm in his voice:

"Vell, doctor, vat did she die of—imbrovements?"

---

An Irishman had appendicitis. They took him to the hospital, laid him on the operating table, gave him ether, and tied a small monkey on a shelf where he would see it when he regained consciousness. He saw it grinning and chattering. "Phat's that?" "Sh—be quiet! That's what we took from you." He uttered a groan and said: "Be ye child or devil, I don't know, but yer mother is a very sick man."

---

The Doctor Who Saved Him.—A story is told of an Englishman who had occasion for a doctor while staying in Pekin.

"Sing Loo gleatest doctor," advised his native servant. "He savee me lifee once."

"Really?" queried the Englishman.

"Yes, me tellible awful." was the reply.

"Me callee in another doctor. He givee me medicine. Me velly, velly bad. Me callee in another doctor. He come and give me more medicine. Make me velly, velly badder. Me callee in Sing Loo. He no come. He savee my life."

---

No Difference.—The stupid person sometimes says a witty thing without knowing it. A professor in a medical college had one exasperating student.

"You see, Mr. Smith," said the professor to this young man one day, "the subject of this diagram limps, because one of his legs is a trifle shorter than the other. Now, what should you do in such a case?"

"I should limp, too, I think, sir," replied the student, with an expression of perfect innocence on his face.—*Sacred Heart Review.*

---

RARE EXPERIENCE.—"I would like to pay a bill," said the caller.

The doctor looked at him curiously for a moment. "Are you quite sure about that?" he asked.

"Why, of course. Here's the money."

"Thanks. I'll write a receipt for you. Pardon my question. You are the first person who ever told me that he would like to pay a bill."

---

THE DOCTOR.—

Ah, who would choose to be a doctor—
A microbe-stalking pill-concocter!
At 3 a. m. they ring his bell
Because some fellow's dined too well.
He has to leave a joyous frolic
Because a baby gets the colic;
And while subduing mortal ills
With jalap, ipecac and squills,
He has to hear the conversations
Of patients, matching operations;
And then, to crown his pain and strife,
They vilify him here, in Life!

—*Arthur Guiterman, in Life.*

---

FREE ADVICE.—A man with the croup halted a doctor on a quiet street corner.

"Doctor," he said, coughing violently, "what ought a chap to do when he's got the croup?"

The doctor's eye emitted a steely light at the thought of being buncoed out of a free prescription, and he said:

"Such a man, my friend, ought to consult a good physician."

"Thanks, doctor," said the sufferer, as he took his leave. "That's what I'll do, then."—*Baltimore American.*

